# Treatment outcome of an intensive psychiatric home treatment for children and adolescents: a non-randomized controlled pilot evaluation

**DOI:** 10.1007/s00787-021-01919-y

**Published:** 2021-12-01

**Authors:** Daniel Graf, Stefan Lerch, Ulrich Böhnke, Corinna Reichl, Jochen Kindler, Julian Koenig, Michael Kaess

**Affiliations:** 1 University Hospital of Child and Adolescent Psychiatry and Psychotherapy, University of Bern, Bern, Switzerland; 2 Department of Child and Adolescent Psychiatry, Center for Psychosocial Medicine, University of Heidelberg, Heidelberg, Germany; 3 Department of Child and Adolescent Psychiatry, Psychosomatics and Psychotherapy, Faculty of Medicine and University Hospital Cologne, University of Cologne, Cologne, Germany

**Keywords:** Home treatment, Treatment setting, Therapy research, Children and adolescents, Child and adolescent psychiatry

## Abstract

Home treatment (HT) may offer an effective and cost-efficient alternative to inpatient treatment for children and adolescents with acute mental disorders. This study introduces and evaluates a pilot HT project from Bern, Switzerland, with HT completely replacing an inpatient treatment. A total of *n* = 133 children and adolescents with acute mental disorders and inpatient treatment needs were treated either in the new HT program (*n* = 37) or in an active control group with inpatient treatment as usual (I-TAU, *n* = 96). Psychopathological burden was assessed by the Health of the Nation Outcome Scale for Children and Adolescents clinician-rated (HoNOSCA) and self-rated (HoNOSCA-SR) at the time of admission and at discharge. Treatment effects were assessed and compared using Augmented Inverse Probability Weights to adjust for baseline differences and to control for treatment duration. Participants ranged in age from 6 to 17 years (*M* = 13.71 years, *SD* = 2.93), 54% were female. HT resulted in significant improvements in the HoNOSCA (*d* = 0.79, *p* < .001) and HoNOSCA-SR (*d* = 0.63, *p* = .006). No significant differences on treatment effects were observed between HT and the reference group I-TAU in the HoNOSCA (*d* = 0.01, *p* = .96) or the HoNOSCA-SR (*d* = 0.11, *p* = .63). Overall, results indicate HT to be an effective alternative for children and adolescents with acute mental health disorders instead of hospitalization. Further evaluation with random group allocation and long-term follow-up should attempt to replicate and extend the current findings.

## Introduction

Mental health disorders in children and adolescents are associated with substantial impairments in various aspects of psychosocial functioning and quality of life [1]. Based on longitudinal data from a large epidemiological sample, Caspi et al. [2] reported that 59% of all participants met criteria for a mental disorder by age 18. In addition, 69% of those who met criteria for at least one mental disorder at age 45 received their first diagnosis by age 18, indicating the particular need for mental healthcare in children and adolescents [2, 3]. Despite the apparent need for treatment services in this age group, children and adolescents with mental health disorders in Switzerland [4], and many other parts of the world [5–7], have impeded access to appropriate and intensive care including inpatient treatment. To address the challenge of an increasing demand for intensive mental healthcare among youth, there is growing interest in developing alternatives that are less costly, yet not less effective than inpatient treatment.

One treatment modality discussed as a promising alternative to conventional inpatient treatment is home treatment (HT) for children and adolescents with mental disorders [8, 9]. HT offers the opportunity to conduct intensive child and adolescent psychiatric treatment using the infrastructure and supervision of the respective family or caregivers. Thus, HT requires fewer resources than inpatient treatment, and allows to strongly involve the patient’s living environment in therapy, which is proposed to reduce the risk of failed transfer of treatment achievements after discharge [10]. The exact operationalization of HT differs across studies, with HT either supplementing and shortening an initial inpatient stay or replacing it entirely. Boege et al. [11] who conducted a randomized controlled trial with HT supplementing hospitalization with *n* = 100 children and adolescents in Germany, reported significant clinical improvements 8 months after the treatment in both the HT and inpatient control group without any group differences. Economic efficiency was significantly better in the HT group [12]. A recent study from the Netherlands also conducted HT supplementing a short hospitalization, and the symptom load of the *n* = 112 participants decreased by over 50% [13]. In a German study (*n* = 105) comparing sole HT to inpatient treatment, Schmidt et al. [14] found that treatment effect was superior in the inpatient group directly after treatment but patients in the HT group showed a more stable maintenance of the treatment effects at 1-year follow-up. Reanalyzing two dissertations from the early 1990s, Mattejat et al. [15] found that there was no difference between the HT and the inpatient control group (*n* = 68) concerning the course of marked psychiatric symptoms and adaptation in school or work after almost four years. Similar results were obtained by Henggeler et al. [16], who investigated the effectiveness of home-based Multisystemic Therapy (MST) in comparison to an inpatient control group (*n* = 113). MST showed better results in a wide range of outcomes, including youth and family functioning [16], fewer mean days of hospitalization and shorter duration of inpatient stay [17] and reduced rates of suicide attempts after one year [18].

Building on this pioneering and promising work, the University Hospital of Child and Adolescent Psychiatry and Psychotherapy (CAP) Bern developed and implemented the HT program “AT_HOME” in May 2019 as an alternative to conventional inpatient treatment [19]. The aim of the program is to establish a new and equally effective treatment service for children and adolescents with acute mental health disorders while reducing treatment costs. Unlike most of the current HT programs reported in the literature, AT_HOME completely replaces an inpatient stay rather than supplementing it. The present pilot data that were obtained for quality assurance of the AT_HOME implementation aimed to compare the treatment outcomes of AT_HOME with conventional inpatient treatment at the CAP.

## Materials and methods

The data presented were derived from a regular quality assurance process, which has been established at the CAP in Bern to evaluate the implementation of AT_HOME. The retrospective use of this data for research purposes was approved by the respective institutional review board (BASEC number: REQ-2020–00546).

### Population and recruitment

Patients included in the analyses were children and adolescents with acute mental disorders who were treated between May 1, 2019 (earliest admission) and July 20, 2020 (latest discharge) either in one of the inpatient units or in the AT_HOME program, both at the CAP Bern. All participants were between 6 and 17 years of age at the time of treatment, living in the canton of Bern in Switzerland, and presented with a mental disorder that resulted in an explicit referral to inpatient treatment.

Patients were included in the AT_HOME program if they lived in a stable housing situation within a 30-min catchment area of the CAP, were able to speak and understand German language, and if written informed consent for participation was obtained from parents and patients over 13 years of age. Exclusion criteria were the presence of acute and severe child welfare hazards in the patient’s household and acute endangerment to self or others that required immediate protection. At first consultation, all patients referred to the CAP for inpatient treatment between May 1, 2019 and May 1, 2020 who met inclusion criteria (*n* = 71) were introduced to the AT_HOME program, and were informed about the possibility to participate in this new treatment program. Interested patients and their families were provided with detailed information about the program and its structure. Subsequently, patients had the opportunity to choose between HT or standard inpatient treatment. Thirty-four patients/families (47.9%) who met the inclusion criteria decided against treatment in AT_HOME. Main reasons cited by parents for refusal were “the family feels overwhelmed” and “stressful family conflicts”. Main reasons given by patients for refusal were “I need distance from the family” and “I believe that I can make faster progress in treatment in the clinic”. A total number of *N* = 133 patients were treated in the defined time period either in AT_HOME (*n* = 37) or in one of the inpatient units of the CAP (*n* = 96). There was one drop out in the AT_HOME sample with a patient prematurely leaving treatment after an aggressive act against a member of the treatment team. All available data were considered for our intention-to-treat analyses.

Due to the retrospective nature of the present study, no a priori power calculation was conducted. However, sensitivity power analyses were carried out to allow post-hoc justification of sample sizes used for this manuscript. With the error probability α set to 0.05 and the predefined sample size of *n*_1_ = 37 and *n*_2_ = 96, a medium difference between groups of *d* = 0.54 can be assumed to be found with a power of 1 – β = 0.8 (two tailed testing).

### Therapeutic interventions

Patients were treated in one of two different treatment conditions (further detailed in Sects. Home Treatment (AT_HOME) and Inpatient treatment as usual (I-TAU), respectively): one group received HT at their residence while the other group received inpatient treatment as usual (I-TAU) in an inpatient unit of the CAP. Irrespective of the condition, all patients completed an intensive psychiatric and psychotherapeutic treatment, which included the support of different health care professionals (i.e., medical doctors, clinical psychologists, social educators and social workers and specialist nurses) and multimodal specialized therapies (e.g., skills training, resource activation, etc.). Based on the overall approach to therapy within the University Hospital, progress of therapy and individual as well as systemic treatment goals were defined according to principles of shared decision-making between patients, caregivers, and therapists in both settings. The goals were clinically monitored thereafter in the form of team meetings (without patients and caregivers) and weekly rounds with the team and patients in the inpatient units and team, patients, and caregivers in AT_HOME.

#### Home treatment (AT_HOME)

The AT_HOME program involves an intensive and inpatient-equivalent form of outreach treatment for children and adolescents aged 6–17 years with acute mental disorders and is financed in part by the health insurance companies and in part by the canton of Bern. Instead of a hospitalization, HT-patients were visited by a member of the treatment team at least once (if needed several times) a day with sessions lasting between 60 and 120 min. On Sundays, the physical contact was often replaced by a telephone call. A maximum of 10 patients and their family could be treated concurrently. Meetings usually took place in the patients’ homes, but could also be arranged at other involved locations such as the patient’s school or workplace. In addition, all families were provided with an acute crisis management phone number where a member of the AT_HOME team could be contacted 24 h/7d. Similar to inpatient treatment, patients received multidisciplinary child and adolescent psychiatric care. Therapy components were closely aligned with those of inpatient treatment and included all interventions that contributed to the reduction of symptoms and improvement of the patient’s and system’s functioning levels. Somatic examinations such as blood sampling or ECG formed also part of the AT_HOME treatment as in I-TAU, but also took place at the patient’s home. Different from inpatient treatment, family members, confidants, and other key individuals (e.g., teachers) were intensively involved in both the treatment and the care of the patients, and patients continued their lives within their families and regular schools. The AT_HOME treatment team cooperated closely with the emergency unit of the CAP so that patients could be hospitalized immediately in the event of acute suicidal tendencies, if 24-h surveillance was required. In the event of an emergency hospitalization, the AT_HOME team continued as the respective treatment team and accompanied the patient during the stay in the emergency unit for a maximum of three days. In case of a longer hospitalization in the emergency unit, the participant was considered a drop-out of AT_HOME. However, this situation did not occur during the pilot evaluation. A suicide attempt was considered a criterion for discontinuation of AT_HOME, as this form of treatment could not provide the intensive surveillance of the patient required in this case. Again, this situation did not occur during the pilot evaluation. Contrary to I-TAU, the duration of treatment in AT_HOME was limited to 3–4 months.

#### Inpatient treatment as usual (I-TAU)

In the I-TAU group, participants received inpatient treatment on one of five inpatient units at the CAP Bern. The CAP Bern is one of Switzerland’s largest institutions for child and adolescent psychiatry and psychotherapy providing mental healthcare for minors within a population of more than one million inhabitants. For the duration of therapy, patients in I-TAU lived with other children and adolescents in their respective unit, participated in the routine clinical program, activities and therapies, and attended the clinic school. Staff was present at any time around the clock, and the duration of treatment was not limited. A descriptive comparison between the treatment conditions is given in Table 1.

**Table 1:** Differences and commonalities between treatment conditions

		AT_HOME	I-TAU
Differences	Setting	Patient at home in family, school, everyday life	Patient in the clinic and clinic school
	Care	At least one visit per day, emergency contact available 24 h/7d	Clinic staff available 24 h/7d
	Duration	Max. 3–4 months	No temporal limitation
	Focus	Patient-environment interaction	Patient
	Inclusion of caregivers	Almost daily family meetings	Weekly family meetings
	Frequency of different treatment components…		
	–Individual therapy with patient	2 / week by psychotherapists	1–2 / week by psychotherapists
	–Individual therapy with caregiver	1 / week by psychotherapists	1 / one to two weeks by psychotherapists
	–Group therapy	–	1 / week by psychotherapists
	-Supportive therapy like empowerment, social competencies training, skills training etc	2–3 / week by social educators and specialist nurses	3–4 / week by social educators and specialist nurses
		AT_HOME	I-TAU
Commonalities	Treatment components	Therapy components comprising interventions to reduce symptoms and improve patient’s and system’s functioning level	
	Target group	Patients aged 6–17 years presenting with a mental disorder that resulted in an explicit referral to inpatient treatment	
	Treatment team	Medical doctors, clinical psychologists, social educators, and specialist nurses	

### Outcome variables and endpoints

Assessments were conducted in both AT_HOME and I-TAU as part of the clinic’s quality assurance process. At the time of admission (baseline) and the end of the treatment (postline), the *Health of the Nation Outcome Scale for Children and Adolescents* (HoNOSCA) [20] was assessed for all patients by clinician ratings and for adolescents ≥ 12 years of age additionally by self-rating (HoNOSCA-SR) [21]. The HoNOSCA is designed to cover a range of behavioral, symptomatic, social, and impairment domains and provides a global outcome for psychopathology in the clinical setting. The scale consists of 13 items answered on a 5-point Likert scale (0 = “not at all” – 4 = “most severe problem”); thus, a higher sum score indicates higher levels of problems present. Clinicians who assessed the HoNOSCA received periodic training to ensure the reliability of the assessment, but were not blinded to treatment condition. Assessments of the HoNOSCA were conducted by four clinicians in the HT group and 17 clinicians in the I-TAU group. Psychometric properties were not calculated in the present study but have been shown to be acceptable in previous studies [21, 22]. Psychosocial functioning was assessed at the time of admission using the *Global Assessment of Functioning Scale* (GAF) [23, 24], which also showed acceptable interrater-reliability in the clinical work with children and adolescents [25]. The GAF is coded on a scale from 1 (no functioning at all) to 100 (perfect functioning). *Functioning* refers to an individual’s ability to manage daily life with all social and role-related responsibilities. Treatment satisfaction was assessed after completion of the treatment by an independent research institute (“B&A – Beratungen und Analysen”; Consulting and Analyses, Bern) with a questionnaire designed by the CAP to assess treatment satisfaction on six different scales. Patients aged 12 years and older and parents of all patients were asked how much they agreed with various statements including “Overall, I am satisfied with the treatment” or “I would recommend the treatment to others” on a 5 point Likert scale ranging from 1 = “not at all satisfied” to 5 = “absolutely satisfied”. Thus, higher scores indicate higher levels of satisfaction. The items on the questionnaire were subsumed into six subscales: “Satisfaction with… 1. Initiation phase, 2. Information and transparency, 3. Treatment phase, 4. Team, 5. Completion phase, and 6. Treatment benefit.” The psychometric characteristics of this questionnaire have not been investigated in previous studies. The internal consistency of the satisfaction questionnaire was very high, with Cronbach’s α = 0.96 for the patient questionnaire and α = 0.95 for the parent questionnaire. The questionnaire in AT_HOME differed slightly from the I-TAU original as some questions about inpatient treatment could not be adapted for HT, such as satisfaction with the menu. In addition, patient and treatment characteristics such as age, gender, clinical diagnosis, and the duration of treatment were drawn from the patients’ medical records.

### Data analyses

In case of homogenous variances between AT_HOME and I-TAU, two-tailed two sample *t* tests were used to investigate group differences for dimensional demographic and clinical variables as well as baseline data, otherwise Welch’s test was conducted. Fisher’s exact test was applied to test group differences for categorical variables. Paired *t* tests were used to compare baseline and postline scores within groups. To compare the two treatments, the average treatment effect was calculated using the propensity score method of Augmented Inverse Probability Weights (AIPW) to adjust for baseline differences between AT_HOME and I-TAU that may have occurred due to the lack of randomization in the recruitment process. AIPW estimators compute the averages of the augmented inverse-probability weighted outcomes for each level of treatment. In a first step, a probit model was employed to predict the treatment group as a function of demographic and baseline data. The parameters of the model were used to compute the inverse probability weight of each patient for assignment to his or her treatment condition. In a second step, linear regressions were employed to model the treatment-specific predicted outcome for each patient, using the baseline data as predictors and controlling for the treatment duration. In a final step, the weighted means of both treatment groups were calculated using the former computed inverse probability weights. The difference of these means represents the average treatment effect. All analyses were conducted in Stata/SE 16.1 except for sensitivity power analyses which were conducted in G*Power v3.1. A *p* value < 0.05 was defined as criteria for statistical significance.

## Results

### Sample characteristics

Of the *N* = 133 patients that were included in the analyses, *n* = 37 patients (16 females) were treated in AT_HOME and *n* = 96 (56 females) in I-TAU. Descriptive statistics of demographic and clinical data as well as baseline characteristics for both groups are depicted in Table 2. No significant group differences regarding sex, age, and HoNOSCA score were found. Significant group differences were found for the GAF score (AT_HOME < I-TAU), the HoNOSCA-SR score (AT_HOME < I-TAU), and distribution of the principal diagnoses.

**Table 2:** Demographic and baseline data of the two treatment groups and the total sample, with means and standard deviations (if not otherwise indicated), and comparison between the two groups

	AT_HOME	I-TAU	Total sample	Test statistics
Age (*mean* ± *SD*)	13.65 ± 2.75	13.73 ± 3.01	13.71 ± 2.93	*t*_131_ = – 0.14, *p* = .89
GAF (*mean* ± *SD*)	40.84 ± 8.14	46.08 ± 10.27	44.41 ± 9.91	*t*_114_ = 2.73, *p* = .01
HoNOSCA (*mean* ± *SD*)	20.65 ± 7.18	21.43 ± 6.45	21.21 ± 6.65	*t*_130_ = 0.61, *p* = .55
HoNOSCA-SR (*mean* ± *SD*)	14.04 ± 8.69	21.9 ± 9.88	19.72 ± 10.52	*t*_75_ = 3.4, *p* < .01
Principal diagnoses (ICD-10)^a^; *n* (%)				χ^2^_6, *n* = 133_ = 19.47, *p* < .01
F1	0	2 (2.08%)	2 (1.5%)	
F2	1 (2.7%)	5 (5.21%)	6 (4.51%)	
F3	5 (13.51%)	29 (30.21%)	34 (25.56%)	
F4	20 (54.05%)	16 (16.67%)	36 (27.07%)	
F6	2 (5.41%)	6 (6.25%)	8 (6.02%)	
F8	3 (8.11%)	16 (16.67%)	19 (14.29%)	
F9	6 (16.22%)	22 (22.92%)	28 (21.05%)	

^a^For a translation of the ICD-10 codes into DSM-5 diagnoses, see DSM-5, Classification section [26, p. xiii ff]

### Treatment effects

#### Pre–post effects of treatment

The descriptive postline statistics for AT_HOME and I-TAU are illustrated in Table 3. Within the two groups, both the HoNOSCA and the HoNOSCA-SR scores decreased significantly, represented by medium to large effect sizes.

**Table 3:** Mean and standard deviation of the postline data (columns t_1_) and difference to baseline (t_0_ – t_1_) split by setting. Effect sizes are depicted as Cohen`s d

	AT_HOME sample				I-TAU sample			
	*n*^a^	*t*_1_	*t*_0_—*t*_1_	Effect	*n*^a^	*t*_1_	*t*_0_—*t*_1_	Effect
HoNOSCA	37	15.27 ± 8.03	5.38 ± 6.84	*d* = 0.79, *t* = 4.79, *p* < .01	95	13.68 ± 6.02	7.75 ± 6.11	*d* = 1.27, *t* = 12.35, *p* < .01
HoNOSCA-SR	23	10.39 ± 7.98	3.78 ± 5.98	*d* = 0.63, *t* = 3.04, *p* < .01	44	13.25 ± 9.36	9.36 ± 9.9	*d* = 0.95, *t* = 6.27, *p* < .01

^a^Cases without missing

#### Comparison of treatment

On average, patients in AT_HOME had a shorter treatment duration than in I-TAU (83.73 ± 27.97 days in AT_HOME vs 100.76 ± 62.70 days in I-TAU; *t*_128.2_ = 2.16, *p* = .04). Controlling for treatment duration, no significant difference was observed in the average treatment effect between AT_HOME and the reference group I-TAU in the HoNOSCA with patients in AT_HOME having an average value of 13.67 and patients in I-TAU of 13.62 (*n*_AT_HOME_ = 37, *n*_I-TAU_ = 95; Δ_AT_HOME—I-TAU_ = 0.05, *d* = 0.01, 95% *CI* = [– 2.18, 2.28], *p* = .96). Similarly, there was no significant difference between conditions in the HoNOSCA-SR with patients in AT_HOME having an average value of 12.86 and patients in I-TAU of 11.94 (*n*_AT_HOME_ = 23, *n*_I-TAU_ = 44; Δ_AT_HOME—I-TAU_ = 0.92, *d* = 0.11, 95% *CI* = [– 2.78, 4.61], *p* = .63). Analyses on group differences remained non-significant even when not adjusting for treatment duration.

### Treatment satisfaction

Considering all families treated in AT_HOME (*n* = 37), 30 parents or pairs of parents (81%) completed the questionnaire on treatment satisfaction as well as did 21 out of the 31 patients aged 12 or older (68%). In the I-TAU group (*n* = 96), 57 parents or pairs of parents (59%) completed the questionnaire as well as did 37 out of the 74 patients aged 12 or older (50%). The response rate was significantly higher in the AT_HOME group for both the parent questionnaire (χ^2^_1, *n* = 133_ = 5.56, *p* = .02) and the patient questionnaire (χ^2^_1, *n* = 105_ = 7.27, *p* < .01). The average responses on satisfaction with different aspects of treatment are depicted in Table 4 for patients and in Table 5 for the parents. No significant differences occurred when comparing AT_HOME and the I-TAU group.

**Table 4:** Satisfaction of patients with different treatment aspects

Satisfaction with…	AT_HOME			I-TAU			Test statistics
	Items	*n*^a^	*Mean*^b^ ± *SD*	Items	*n*^a^	*Mean*^b^ ± *SD*	
Initiation phase	1	21	3.90 ± 1.18	2	37	4.22 ± 0.67	*t*_56_ = – 1.34, *p* = .19
Information and transparency	7	20	4.20 ± 0.64	7	37	4.00 ± 0.69	*t*_55_ = 1.09, *p* = .28
Treatment phase	4	20	3.91 ± 0.64	6	34	4.02 ± 0.79	*t*_52_ = – 0.56, *p* = .58
Team	9	21	4.43 ± 0.56	9	36	4.30 ± 0.75	*t*_55_ = 0.71, *p* = .48
Completion phase	3	21	3.93 ± 0.89	3	37	4.01 ± 1.08	*t*_56_ = – 0.31, *p* = .76
Treatment benefit	5	21	3.82 ± 0.99	5	33	4.07 ± 1.07	*t*_52_ = – 0.85, *p* = .40

^a^Cases without missing

^b^Scale from 1 = “not at all satisfied” to 5 = “absolutely satisfied”

**Table 5:** Satisfaction of parents with different treatment aspects

Satisfaction with…	AT_HOME			I-TAU			Test statistics
	Items	*n*^a^	*Mean*^b^ ± *SD*	Items	*n*^a^	*Mean*^b^ ± *SD*	
Initiation phase	2	28	4.66 ± 0.50	3	54	4.42 ± 0.58	*t*_80_ = – 1.88, *p* = .06
Information and transparency	8	29	4.42 ± 0.67	9	55	4.39 ± 0.66	*t*_82_ = 0.23, *p* = .82
Treatment phase	5	28	4.33 ± 0.63	6	55	4.29 ± 0.65	*t*_81_ = – 0.31, *p* = .75
Team	6	29	4.76 ± 0.48	6	56	4.66 ± 0.52	*t*_83_ = 0.84, *p* = .40
Completion phase	2	28	4.38 ± 0.76	2	56	4.13 ± 1.03	*t*_82_ = 1.10, *p* = .28
Treatment benefit	7	26	4.20 ± 0.63	7	54	4.13 ± 0.75	*t*_78_ = 0.37, *p* = .71

^a^Cases without missing

^b^Scale from 1 = “not at all satisfied” to 5 = “absolutely satisfied”

## Discussion

This study investigated the treatment outcomes of AT_HOME as a potential and equally effective alternative to inpatient treatment for children and adolescents with acute mental disorders. Using the HoNOSCA and HoNOSCA-SR as indicator of psychopathology burden, we found significant post-treatment improvement in patients treated in AT_HOME with moderate to large effect sizes. Within a non-randomized design, we found no differences in treatment effects between AT_HOME and an I-TAU control group.

Although assignment to treatment in the current study was non-randomized, the two groups did not differ in their demographic characteristics; there were no differences in the distribution of age or gender. However, distribution of primary diagnoses across the two groups differed: in AT_HOME there was a higher proportion of F4 codes (neurotic, stress-related, and somatoform disorders), and patients in I-TAU had higher proportions of F3 and F8 codes (affective disorders and disorders of psychological development; diagnoses according to ICD-10) [27]. These differences probably result from the non-randomized sampling method. Since patients and their families were free to choose whether or not to participate in AT_HOME, patients with anxiety disorders may have preferred to stay at home for therapy rather than go to the hospital. In contrast, families of patients with affective disorders, such as major depression, possibly hoped for an activating effect from the daily structure provided on the inpatient unit and felt overwhelmed with the idea of structuring daily life in the home environment. Also, fear of suicidal acts is common in families of patients with affective disorders, so they may have preferred inpatient treatment for around-the-clock supervision. In comparison to previous HT studies [11, 14, 16, 28], our sample showed reduced rates of externalizing disorders. This may be due to the fact that some of the previous HT studies—i.e., trials with MST—have focused on the treatment of conduct disorders [28, 29] or have defined certain diagnoses as exclusion criteria for HT [30]. This resulted in higher rates of externalizing disorders in contrast to our sample, in which no diagnoses were excluded. Compared to studies that included all diagnoses, the proportion of externalizing diagnoses is more similar [11, 13, 31], though still slightly lower in AT_HOME. This seems plausible, considering the age distribution in the AT_HOME sample was skewed toward older age; only 6 patients (16%) were 11 years or younger. In this age group, F9 codes of the ICD-10 (behavioral and emotional disorders with onset usually occurring in childhood and adolescence) are more common than in older patients. As F9 codes make up a large proportion of the externalizing disorders, this category is underrepresented in our sample. In addition, mood disorders (F3) were slightly underrepresented and anxiety disorders (F4) were overrepresented, compared with other HT studies. This should be taken into account when comparing our findings with those of other HT studies, as patients with different disorders may respond differently to HT [30, 32, 33].

Patients in the two treatment groups differed significantly with respect to their levels of psychosocial functioning at baseline, with decreased functioning in AT_HOME patients. This difference was not expected, as both conditions were tailored to the same group of patients. However, the finding suggests that the AT_HOME sample consisted of patients who were not less impaired in their functioning than those treated in inpatient wards. This is an important aspect, because it contradicts the concern that AT_HOME might “snatch away” less impaired patients from the inpatient units.

In general, clinicians (HoNOSCA) and patients (HoNOSCA-SR) had little agreement (*r* = 0.19, n.s.) in their appraisal of the level of psychopathological burden, as reflected in the mean group differences: although patients did not differ between groups at treatment admission in terms of clinician-rated psychopathological burden (HoNOSCA), patients’ self-rating of HoNOSCA-SR differed significantly between groups. Patients in the AT_HOME group rated their psychopathological burden as less severe than patients in the I-TAU group and as less severe than the respective clinician. The discrepancy between HoNOSCA and HoNOSCA-SR in the HT group could be a methodological artifact, as the HoNOSCA-SR was completed only by patients aged 12 years or older, whereas the HoNOSCA was assessed for all patients regardless of age. One might assume interaction effects in which the older patients were generally less impaired if they could stay at home and the younger patients were the more impaired. However, reanalysis of the HoNOSCA and GAF, which included only patients aged 12 years and older, did not change the results. Maybe patients who were more withdrawn (i.e., with anxiety disorders) received lower ratings of psychosocial functioning and higher ratings of psychopathology on the external assessment but did not feel, or rate themselves, so impaired when given the opportunity to remain at home in their familiar environment and “safe base”. A further explanation is a possible selection bias: patients who subjectively felt less ill were perhaps more confident in seeking treatment in their own home environment and therefore primarily chose this setting. In addition, timing of the assessment should be considered, which was immediately after admission. While patients in AT_HOME experienced relatively little change in their daily routines due to the start of therapy, patients referred to a hospital ward entered a completely different setting. They moved into a new environment with regard to housing, peers, and school. Patients who were asked how they were doing at that moment felt probably more insecure and impaired than patients in AT_HOME who did not experience these changes.

The unadjusted effect sizes of symptom reduction in AT_HOME found in the present study are comparable to previous studies that used HT as a full replacement for inpatient treatment [14, 29]; however, studies using HT as an supplement to inpatient treatment found slightly higher effect sizes that are comparable to those of our I-TAU [11, 13]. During inpatient treatment, children and adolescents are temporarily removed from their often problematic environment and relieved of the stress potentially associated with the family or school setting. This temporary relief might be one reason why in the present study the I-TAU condition led to a slightly higher reduction in psychopathology burden than AT_HOME, when descriptively compared.

However, the unadjusted treatment effect of the two treatment conditions cannot be directly compared because allocation to the groups was non-randomized and systematic group differences occurred at baseline, as observed for the HoNOSCA-SR. We employed AIPW-analyses to account for these differences and to control for treatment duration. We chose the inverse probability method because there was insufficient overlap between the two groups for propensity score matching, too few data were available for stratification, and AIPW better resemble an RCT compared with regression-adjustment of baseline data alone. Calculation of the adjusted treatment effect using AIPW models yielded a null effect for differences between the AT_HOME and I-TAU groups on clinician-rated and self-rated psychopathological burden, although the unadjusted effect sizes differed. These results are consistent with previous studies that found no differences in treatment outcomes for HT compared with an I-TAU control group [11, 15, 16, 34]. There is evidence in the literature that treatment effects achieved with HT may remain more stable than those achieved with I-TAU [12, 14]. Improvements achieved during HT are directly incorporated into the patient’s daily life, which prevents the risk of failed transfer after discharge. To evaluate the stability of the treatment effect achieved, a follow-up of the present sample seems critical for the future.

One should keep in mind that patients and their families in AT_HOME had to make an active choice to receive HT, unlike most patients in the control group, who did not meet the inclusion criteria and therefore could not choose which treatment they wanted. As the expectation towards treatment may have influenced treatment choice, participants in AT_HOME may have had more positive expectations towards the upcoming treatment. Positive treatment expectation in turn has been shown in previous studies to increase patient and parent adherence to treatment, leading to better treatment outcomes and higher patient satisfaction [35–37]. It is, therefore, possible that treatment effects and satisfaction in the HT group were slightly overestimated compared with the I-TAU group.

Treatment satisfaction among patients and families in AT_HOME was generally high. For example, 71% of patients and 85% of parents indicated to rather agree or to agree completely with the statement “*Overall, I am satisfied with the treatment*”, while no one disagreed. With the statement “*I would recommend the treatment to others*”, 85% of patients and 87% of parents indicated to rather agree or to agree completely, while 5% of patients and parents agreed rather not or not at all. On average, patients showed slightly lower satisfaction than parents, which is consistent with results of previous studies on HT among children and adolescents [16, 38, 39]. Comparing the satisfaction data of patients treated in AT_HOME with those in the I-TAU group revealed no relevant differences, indicating comparable subjective benefits of patients in both groups. Response rates for the questionnaire on treatment satisfaction were significantly higher in the AT_HOME group than in the I-TAU group. Similar patterns in the response rate of satisfaction questionnaires have been reported previously [39] and might reflect a higher adherence to clinical guidelines by the treatment team in the new treatment condition, which more actively encouraged patients to respond to the satisfaction questionnaire. Another possibility is response bias, as treatment satisfaction has been shown to correlate with response rate [40], possibly overestimating treatment satisfaction in both groups, with greater overestimation in the I-TAU group.

An interesting aspect concerns the application of HT in pandemic situations. A large body of literature has investigated the impact of the March 2020 COVID-19 pandemic outbreak on young people and their families [e.g., 41]. As summarized by UNICEF in a recent report, school closures, home office, and the loss of social networks resulted in considerable distress for many families at home and a significant increase in mental illness among young people, warranting special support [42]. At the same time, many mental health services had been closed due to quarantine regulations. The AT_HOME project was designed and implemented before the COVID-19 pandemic outbreak in March 2020, and most of the data presented in this article were collected before this time. Therefore, no direct conclusions can be drawn about the adequacy of HT in the context of a pandemic. However, we suggest that HT could offer an important component in supporting both young patients with mental health issues and their family systems during the pandemic, as problems arising from the new situation can be directly observed and addressed in the respective environment. Also, patients in HT can be treated independently of other patients. A positive COVID-19 test may imply the quarantine of an entire inpatient unit, which is not the case in HT, where the team can continue to visit the remaining patients.

## Limitations and strengths

The current study has some limitations. First, finding no differences between the two conditions does not automatically preclude the absence of real differences [43]. The current analyses were robust against false positive results but do not ensure against false negative results. We performed sensitivity power analyses, which showed that we could expect to find medium group differences of *d* = 0.54 with high statistical power of 1 – β = 0.8 based on the present sample size. It is possible that small differences exist between the two conditions that could not be detected due to the limited sample size. However, this seems rather unlikely because all effects found for differences between groups were minimal. The current data provide no evidence to reject the hypothesis that the two interventions have the same effect.

Second, we had limited data restricted to the assessment procedure prescribed by the mandatory Swiss ANQ initiative [44] which defines the clinical routine. HoNOSCA and GAF were not rated by independent researchers but by clinical staff who were not blinded to treatment condition. However, we expect no systematic bias between the groups due to the lack of blinding. In both groups, therapists rated treatment outcome for their own patients which may at least have led to comparable bias in the two groups. Also, using information from different sources with clinician-rated assessments and self-rated assessments of the HoNOSCA(-SR) provides a more comprehensive picture of the actual situation regarding psychopathological burden. GAF data were available only for admission and thus could not be used for the evaluation of the treatment trajectory. HoNOSCA(-SR) data were available only for admission and discharge and do not allow statements about long-term outcomes. In future, follow-up assessments would be important to evaluate the stability of treatment effects.

Third, this was a non-randomized study-design with allocation by choice. Systematic differences between the two treatment groups might have occurred, which limits the external validity of the results. For example, it is likely that patients with anxiety disorders generally prefer HT to treatment in the clinic, resulting in an overrepresentation of these patients in the AT_HOME group. Another consequence of the non-randomized design was the uneven distribution of patient numbers in the two groups. The I-TAU group pooled patients from five different inpatient units of the CAP and therefore was considerably larger than the HT group, which was composed only of patients from the AT_HOME program. However, the analyses revealed that the two treatment samples were similar in terms of their demographics and most baseline data. Further evaluation with randomized assignment to treatment condition would be desirable to support the current findings and increase their external validity.

A particular strength of the present study is the stringent operationalization of HT as a full replacement for inpatient treatment. This allows us to draw a clear conclusion concerning the efficacy of the HT program, in contrast to most previous studies that used HT as a supplement to inpatient treatment, which makes it difficult to disentangle the treatment effect of HT from the supplemented inpatient treatment.

The recruitment process followed a rigorous procedure. Participation in AT_HOME was only offered to patients referred to the CAP for inpatient treatment, which ensured that only patients who would have been treated in a clinical inpatient ward were included. At the same time, there were virtually no exclusion criteria for clinical diagnoses that could be treated in AT_HOME, resulting in the inclusion of general psychiatric patients with different diagnoses and inpatient treatment needs, which strengthens the external validity of our results. Though relatively small, the composition of the current sample may provide an indication of which patient groups are more likely to choose HT after standard clinical implementation of the HT program, when patients are free to choose their preferred treatment setting.

## Conclusion and implications

With the present study, we aimed to investigate the clinical outcome of a new inpatient-replacing HT for children and adolescents with acute mental disorders. We found a significant reduction in psychopathological burden in patients treated in AT_HOME with no differences in the average treatment effect between the AT_HOME group and an I-TAU control group. These initial results suggest that AT_HOME may be an effective alternative for children and adolescents with acute mental health disorders who would have previously been treated as inpatients. Further research with larger sample sizes and random group assignment should attempt to replicate and extend the current findings. Subgroup-analyses are needed to determine whether there exist certain clusters of patients who benefit more from AT_HOME than others. Future follow-up assessments of the present sample are needed to evaluate the stability of the treatment effects achieved. In the long run, the program could be integrated into the routine health care system in Switzerland as a possible alternative to inpatient treatment, thus driving the shift from treatment in the clinic to treatment AT_HOME.

## Data Availability

Not applicable.
